# Comparative analysis of secreted protein evolution using expressed sequence tags from four poplar leaf rusts (*Melampsora *spp.)

**DOI:** 10.1186/1471-2164-11-422

**Published:** 2010-07-08

**Authors:** David L Joly, Nicolas Feau, Philippe Tanguay, Richard C Hamelin

**Affiliations:** 1 Natural Resources Canada, Canadian Forest Service, Laurentian Forestry Centre, 1055 du PEPS, P.O. Box 10380, Stn. Sainte-Foy, Québec, QC, G1V 4C7, Canada; 2 Department of Forest Sciences, Faculty of Forestry, University of British Columbia, Vancouver, BC, V6T 1Z4, Canada; 3 Unité Mixte de Recherche 1202, Institut National de la Recherche Agronomique-Université Bordeaux I, Biodiversité, Génes et Communautés (BioGeCo), INRA Bordeaux-Aquitaine, 33612 Cestas Cedex, France

## Abstract

**Background:**

Obligate biotrophs such as rust fungi are believed to establish long-term relationships by modulating plant defenses through a plethora of effector proteins, whose most recognizable feature is the presence of a signal peptide for secretion. Since the phenotypes of these effectors extend to host cells, their genes are expected to be under accelerated evolution stimulated by host-pathogen coevolutionary arms races. Recently, whole genome sequence data has allowed the prediction of secretomes, facilitating the identification of putative effectors.

**Results:**

We generated cDNA libraries from four poplar leaf rust pathogens (*Melampsora *spp.) and used computational approaches to identify and annotate putative secreted proteins with the aim of uncovering new knowledge about the nature and evolution of the rust secretome. While more than half of the predicted secretome members encoded lineage-specific proteins, similarities with experimentally characterized fungal effectors were also identified. A SAGE analysis indicated a strong stage-specific regulation of transcripts encoding secreted proteins. The average sequence identity of putative secreted proteins to their closest orthologs in the wheat stem rust *Puccinia graminis *f. sp. *tritici *was dramatically reduced compared with non-secreted ones. A comparative genomics approach based on homologous gene groups unravelled positive selection in putative members of the secretome.

**Conclusion:**

We uncovered robust evidence that different evolutionary constraints are acting on the rust secretome when compared to the rest of the genome. These results are consistent with the view that these genes are more likely to exhibit an effector activity and be involved in coevolutionary arms races with host factors.

## Background

Rust fungi or *Pucciniales *(= *Uredinales*) represent the largest group of fungal plant pathogens, including more than 7000 species that possess the most complex life cycles in the Kingdom Fungi [1]. Some of these obligate biotrophs have been of long standing concern for agriculture and forestry while others have emerged in recent epidemics. For instance, poplar leaf rusts belonging to the genus *Melampsora *are considered as the world's most important disease of poplars [2]. Selection for durable resistance to these pathogens is thus an important challenge for poplar breeders [3]. Although poplar breeding programs have been in place for decades in Europe, clones selected for complete resistance against rust have increasingly succumbed in time to new races of *Melampsora larici-populina *[4]. Sustainability of newly selected resistance clearly requires a better understanding of the molecular mechanisms involved in *Populus*-*Melampsora *interactions.

Prokaryotic and eukaryotic plant pathogens have evolved highly advanced strategies to engage their hosts in intimate contacts and deliver suites of effector proteins to modulate plants' innate immunity and enable parasitic colonization [5-12]. Understanding the translocation mechanisms of bacterial pathogen effectors inside host cells has been an outstanding breakthrough with the characterization of the type III secretion system. This export apparatus enables a bacterium to manipulate host cellular processes by injecting effector proteins into the host cytoplasm [7]. Similarly, plant-parasitic nematodes have developed diverse relationships to obtain nutrients from their host plants. Perhaps their most evolutionary sophisticated adaptations are effector proteins encoded by parasitism genes expressed in oesophageal gland cells and secreted through a protrusible feeding spear, called a stylet [5,6]. However, still little is known about such translocation machineries in filamentous pathogens (mainly fungi and oomycetes), although another specialized biotrophic infection cell called the haustorium is thought to be involved [13-15]. The haustorium invaginates host cells and makes near-direct contact with the host plasma membrane, where it plays a crucial role in nutrient acquisition. This structure is a regulatory hub involved in the manipulation of host metabolism and the suppression of host defenses, which allows the establishment of a successful biotrophic relationship [16-19]. The concomitance of haustoria formation with the induction of a programmed cell death response termed the hypersensitive response (HR) suggests a significant role for this structure in delivering effector proteins into the infected host cell [14,20].

Key insights have emerged from the recent identification of filamentous pathogen effectors with avirulence activity inducing plant defense responses and HR [21-26]. Most of the avirulence genes identified encode small proteins with N-terminal signal peptides that direct them through the endoplasmic reticulum secretory pathway [14,27]. While effector genes reside in pathogen genomes, their products essentially generate phenotypes that extend to host cells and tissues, and are hence likely to be the direct target of the never-ending coevolutionary conflict between host and pathogen [28,29,36]. In fact, avirulence proteins recognition by plant resistance proteins imposes selection against effector function, and pathogen effector proteins probably overcome resistance through diversification of the genes encoding them [30]. For instance, several avirulence genes or their plant counterparts display molecular hallmarks of positive selection [21,22,25,30-38]. Recently, the availability of filamentous plant pathogen genome sequences facilitated the cataloguing of whole secretomes using computational analyses, thus allowing the identification of putative effectors [39-43]. Indeed, Tyler et al. [41] provided evidence that secreted proteins have been subject to accelerated evolution by contrasting the genome sequences of *Phytophthora ramorum *and *Phytophthora sojae*.

Here we provide an overview of the expressed secretome of poplar leaf rusts belonging to the genus *Melampsora*. We constructed cDNA libraries for four related poplar leaf rust pathogens with different host specificities to test for the signature of selection in these rust secretomes and used computational tools to annotate putative secreted proteins. A comparative genomics approach based on homologous gene groups (HGGs) was undertaken to evaluate the extent to which secretome members are under different evolutionary constraints. We describe adaptive evolution (positive selection) in genes encoding secreted proteins of poplar leaf rusts, in agreement with the idea that these genes are expected to display an effector activity and be involved in the escalating and reciprocal coevolutionary arms race with host resistance factors.

## Results and Discussion

### Defining the poplar leaf rust secretome

We constructed cDNA libraries from *ex planta *material of four different *Melampsora *taxa with different host specificities: the Eurasian *M. larici-populina*, the North American *M. occidentalis*, and two *formae speciales *of the North American *M. medusae*, *M. medusae *f. sp. *deltoidae *and *M. medusae *f. sp. *tremuloidae *(Table 1; see Methods). This allowed the comparison of expressed sequence tags (ESTs) with their putative orthologues and made it possible to incorporate evolutionary information into our analyses. In order to extend our dataset with candidate effectors expressed in and secreted from haustoria, we generated an additional haustorium-enriched library (biotrophic stage) of *M. larici-populina*. In total, 14,904 clones were sequenced in this study, which represented 6,044 unique sequences (unisequences, i.e. all contigs and singletons). Clone sequences are available under GenBank accession numbers GW672673 to GW687576. The identification of transcripts coding for secreted proteins was carried out using an *in silico *analysis including a series of prediction algorithms (SignalP, TargetP and TMHMM), yielding 405 sequences encoding putative proteins with predicted secretory signal peptides (# Putative S, Table 1). A number of sources exist that lead to false prediction, i.e. selecting individual proteins that should not be included in the secretome. For example, mitochondrial localization sequences and N-terminal transmembrane anchors are frequently interpreted as signal peptides [44]. Even though no computational method seems fully accurate, the prediction algorithms included in our approach should have excluded such false positives. The assignment of a protein to the secretome is also totally dependent on having the full-length open reading frame (ORF). An inaccurate *ab initio *gene prediction or an incomplete ORF could lack an additional sequence encoding a transmembrane domain or a signal peptide. For this reason, we conducted reciprocal blast between libraries to reduce the number of false assignments due to partial or mispredicted ORFs. Most reassignments were false negatives with truncated N termini, thus increasing the number of putative secreted members to 689 (# Putative S+, Table 1).

**Table 1:** Summary of *Melampsora *cDNA libraries characteristics

Species/Natural host	Material	Unisequences	Contigs	Singletons	Putative S^a ^(%)	Putative S+^b ^(%)
*M. larici-populina*/*Populus nigra*	Haustoria	1148	427	721	54 (4.7)	72 (6.3)
*M. larici-populina*/*P. nigra*	*Ex planta*	787	266	521	51 (6.5)	92 (11.7)
*M. medusae *f. sp. *deltoidae*/*P. deltoides*	*Ex planta*	937	452	485	95 (10.1)	131 (14.0)
*M. medusae *f. sp. *tremuloidae*/*P. tremuloides*	*Ex planta*	1547	571	976	98 (6.3)	246 (15.9)
*M. occidentalis*/*P. trichocarpa*	*Ex planta*	1625	493	1132	107 (6.6)	148 (9.1)

^a^S: Initial predicted set of unisequences encoding putative secreted proteins.

^b^S+: Final set of unisequences encoding putative secreted proteins, following reassignments based on reciprocal BLAST.

### Functional annotation of the poplar leaf rust secretome: novel proteins and the identification of orthologues

**Figure 1:**
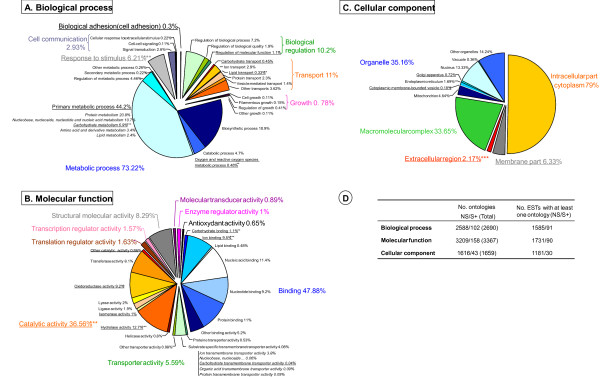
**Gene Ontology classification of the *Melampsora *unisequence dataset**. For each main GO category (A: Biological process; B: Molecular function; and C: Cellular component), percentages were based on the total number of ontologies found for unisequences encoding putative secreted proteins (S+) or non-secreted proteins (NS) (see values in D). Note that individual GO categories can have multiple mappings resulting in percentage values higher than 100%. Underlined GO categories are overrepresented in S+ compared with NS (***: significant at the 0.1% level; **: significant at the 1% level; *: significant at the 5% level).

This S+ dataset was used as search query in BLASTX on the non-redundant UniProtKB database to find sequences with significant matches. Furthermore, to develop an understanding of how protein secretion by poplar leaf rusts might be related to specialized functions or processes, we used the PFAM [45] and Gene Ontology (GO) [46] databases to determine whether any class of proteins was more likely to be found in the *Melampsora *secreted proteins. In addition, we used the Gene Ontology methodology within a statistical framework to determine whether any GO terms were significantly enriched in S+ proteins when compared to non-secreted proteins (NS). For 248 candidates, i.e. 36% of the S+ dataset, significant similarity was found in the UniProtKB database, mostly among putative effectors or proteolytic and carbohydrate-degrading enzymes from other fungal species (Table 2). Less than a quarter of the unisequences had similarity to one or more PFAM domain predictions or GO term assignments. The most abundantly represented PFAM domains in the poplar leaf rusts secretome are presented in Additional File 1, and GO classifications are depicted in Figure 1.

**Table 2:** Similarity of *Melampsora *unisequences to sequences from UniProtKB, BasidiomycotaDB and PuccinialesDB

Library	% with homologues in UniProtKB		% with homologues in BasidiomycotaDB		% with homologues in PuccinialesDB	
	**NS^a^**	**S+^b^**	**NS**	**S+**	**NS**	**S+**
	
*M. larici-populina *haustoria	22.6	13.9	21.5	13.9	29.6	23.6
*M. larici-populina ex planta *material	54.0	54.3	54.1	50.0	62.4	56.5
*M. medusae *f. sp. *deltoidae ex planta *material	56.2	50.4	54.7	45.8	64.9	56.5
*M. medusae *f. sp. *tremuloidae ex planta *material	36.7	23.6	38.3	23.2	47.4	35.4
*M. occidentalis ex planta *material	47.4	43.9	49.3	37.2	59.6	54.7

BasidiomycotaDB included *Basidiomycota *(excluding *Pucciniales*) EST sequence 6-frame translations and protein sequences from the non-redundant database of NCBI and gene models from *Coprinus cinereus *Okayama 7 (#130), *Cryptococcus neoformans *var. *grubii *serotype A, strain H99, *Laccaria bicolor *S238N-H82, *Malassezia globosa *CBS 7966, *Phanerochaete chrysosporium *RP78, *Postia placenta *MAD-698, *Sporobolomyces roseus *IAM 13481 and *Ustilago maydis *521. PuccinialesDB included *Pucciniales *EST sequence 6-frame translations and protein sequences from the non-redundant database of NCBI and gene models from *P. graminis *f. sp. *tritici *CRL 75-36-700-3. BLASTX hits were considered significant when E-value ≤ 1e-4.

^a^NS: Final set of unisequences not predicted to encode putative secreted proteins.

^b^S+: Final set of unisequences encoding putative secreted proteins, following reassignments based on reciprocal BLAST.

Host infection by biotrophic fungi is believed to involve the secretion of effectors that suppress plant defenses and alter cellular metabolism to fulfill the requirements of the invading pathogen [47]. Among the candidates that had significant similarity to known sequences, homologies with potential effectors previously identified in the haustoria of other rust species were observed. Interestingly, 22 candidates had similarities with eight "haustorially expressed secreted proteins" (HESPs) from *Melampsora lini *identified using a similar bioinformatics approach. Some of these HESPs were shown to co-segregate with known avirulence genes and are proven HR elicitors in flax, such as the avirulence protein AvrM [26]. The percentage of identity between flax rust and poplar leaf rust HESPs differed greatly. For instance, HESP-379 had close homologues in *Melampsora *spp. (identity: 93%), while AvrM appeared less conserved (identity: 26%). Other candidates had similarities with Rust Transferred Protein (RTP1) from *Uromyces fabae*, a small secreted protein that is specifically expressed in broad bean rust haustoria and translocated into host cells. This protein accumulates within the cytoplasm of the infected host cell and in the host nucleus, suggesting a role in influencing host gene expression [13]. Homologies with other proteins thought to contribute to pathogenesis were unravelled, with 19 unisequences having similarity to CFEM domain proteins (CFEM = Common in Fungal Extracellular and Membrane). This particular domain is an eight cysteine-containing domain for which members are proposed to have important roles in fungal pathogenesis [48], and it was by far the most highly represented PFAM domain in the S+ dataset. CFEM-containing proteins could function as cell-surface receptors, signal transducers, or adhesion molecules in host-pathogen interactions [48]. Moreover, five unisequences had significant similarity to *gEgh16/gEgh16 H *proteins from *Blumeria graminis*, a large family potentially involved in host-pathogen interactions [49]. An appressorium-specific expression pattern was described for numerous *gEgh16/gEgh16 H *homologues, including virulence genes *GAS1 *(*MAS3*) and *GAS2 *(*MAS1*) from *Magnaporthe grisea*. *GAS1 *or *GAS2 *deletion mutants had no defect in vegetative growth, conidiation or appressoria formation, but were reduced in appressorial penetration and lesion development [50].

The diverse ecological niches of fungal species are mirrored in their secretome, which includes gene families encoding various proteolytic and carbohydrate-degrading enzymes known to act on the linkages found in plant cell walls and compatible with the array of nutritional sources they can exploit [47]. Consistent with the molecular function GO analysis (Figure 1), 36.56% and 47.88% of *Melampsora *secreted proteins with a GO assignment were involved in binding (GO:0005488) and catalytic activity (GO:0003824), respectively. Note that there was a trend towards concentration of a distinct set of processes and functions in the group of proteins making up the *Melampsora *secretome. Significantly higher proportions of secreted proteins, relative to the entire dataset, were assigned to the following functions: carbohydrate and ion binding, and hydrolase and oxidoreductase activity (GO:0030246 and GO:0043167, and GO:0016787 and GO: 0016491). There also appeared to be enrichment of proteins involved in processes related to cell adhesion (GO:0007155), response to stimulus (GO:0050896), regulation of molecular function (GO:0065009), carbohydrate and lipid transport (GO:0008643 and GO:0006869), and primary metabolic process (GO:0044238), including oxygen and reactive oxygen species metabolic process (GO:0006800) and carbohydrate metabolic process (GO:0005975). Apart from putative effectors, members of the secretome had homology to a battery of glycoside hydrolases and subtilisin-like serine proteases that likely contribute to the penetration of the plant cuticle and cell wall [47]. In fact, glycosyl hydrolase 16 (PF00722) was the second most represented PFAM domain in the secretome, followed by domains typically found in proteolytic enzymes (Peptidase_S8 [PF00082], Subtilisin_N [PF05922] and Asp [PF00026]) (Additional File 1). A class of secreted proteins exhibiting the ability to neutralize reactive oxygen species (ROS), and including Mn and Cu/Zn superoxide dismutases, was also uncovered by this survey. This finding was not surprising as it is known that rust fungi prevent a variety of non-specific defense responses in invaded cells, thus allowing the establishment of the long-term biotrophic relationship between rust fungi and living host cells [51]. Such host responses frequently involve the production of ROS, whose detoxification is essential for the establishment of the pathogen. A Mn superoxide dismutase homologue had previously been reported in the haustorial stage of *Puccinia triticina *[52] and was differentially-expressed in *Uromyces appendiculatus *germlings during early appressorium development [53]. Concordant with these observations, previous studies have demonstrated the upregulation of several host genes encoding enzymes of the redox regulation pathways during *Populus*-*Melampsora *interactions [54,55]. Another group of interesting secreted proteins identified here that may be critical for evading host recognition or protecting fungal cell wall from hydrolysis by host enzymes were chitin deacetylases, which have already been described in libraries from *Phakopsora pachyrhizi *[56] and *P. triticina *[52]. The conversion of chitin into chitosan by de-*N*-acetylation not only protects fungal infection structures from hydrolytic attack by chitinases present in the host tissue, it also prevents the release of chitin oligomers responsible for the triggering of resistance reactions [57].

This functional annotation established the secretome's ability to perform diverse roles in pathogenicity and interactions with host cells. However, for the major part of the secretome, no GO or PFAM domains could be assigned. For 441 candidates, i.e. 64% of the S+ dataset, no significant similarity was found to known proteins in the UniProtKB database (E-value > 1e-4) (Table 2). To ascertain that the large number of unmatched proteins identified in this study was not due to the paucity of *Pucciniales *(or even *Basidiomycota*) sequences in international databases [52], we constructed two specific databases (BasidiomycotaDB and PuccinialesDB). BLASTX searches of the complete dataset of *Melampsora *unisequences (S+ and NS) were performed against these databases. The percentage of unisequences with homologues was similar between BasidiomycotaDB and UniProtKB, indicating that the latter is not deficient in *Basidiomycota *sequences (Table 2). As previously observed with BLASTX searches, the percentage of S+ unisequences with homologues in both BasidiomycotaDB and PuccinialesDB was generally lower compared to NS. These results are consistent with the view that secreted effector proteins that subvert host-cell structure and functions often show very limited phylogenetic distribution and no obvious conserved motifs, being less evolutionarily conserved. Furthermore, the percentage of homologues for the haustorium-enriched library was surprisingly low considering that the whole gene set of another rust, *Puccinia graminis *f. sp. *tritici*, was included in the database. However, these results were comparable to observations made on the haustorial secretome of other rust fungi [26,58]. Most of the proteins secreted from the haustorium could be under rapid evolution and strong diversification due to host selective pressures and, therefore, be species-specific. Taken together, these results strongly indicate that a large portion of the secretome (and especially the haustorial secretome) of *Melampsora *may have virulence functions or be involved in host-pathogen interactions.

### Expression profiles of the poplar leaf rust secretome: stage-specificity

**Figure 2:**
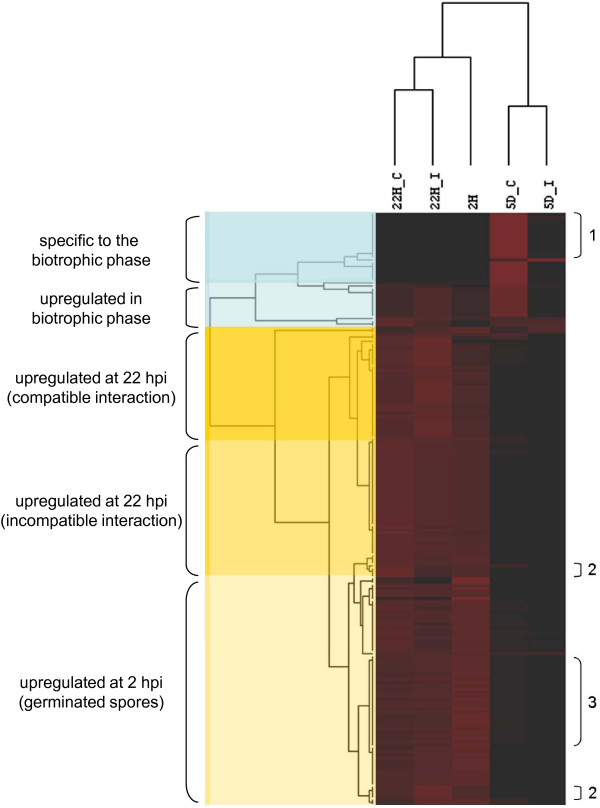
**Serial analysis of gene expression: stage-specificity of putative secretome members of the *Melampsora *unisequence dataset**. Similarities in serial analysis of gene expression (SAGE) patterns of 182 putative secretome members of the *Melampsora *unisequence dataset were determined using an average linkage hierarchical clustering according to the Spearman Rank Correlation. Corresponding tags were identified as described in the Methods section. Each row represents a tag, whereas each column corresponds to a SAGE library. 22H_C: 22 hours after inoculation (compatible interaction); 22H_I: 22 hours after inoculation (incompatible interaction); 2H: 2 hours after inoculation (germinating spores); 5D_C: 5 days after inoculation (compatible interaction); and 5D_I: 5 days after inoculation (incompatible interaction). Genes upregulated during the biotrophic or pre-biotrophic stages are highlighted in blue and yellow, respectively. Medium blue = genes specific to the biotrophic stage; Light blue = genes upregulated during the biotrophic stage; Dark yellow = genes upregulated in 22H_I; Medium yellow = genes upregulated in 22H_C; and Light yellow = genes upregulated in 2 H. The relative abundance of the SAGE tag in the library correlates with the intensity of the red color (black, not present; intense red, highly abundant). Brackets indicate clades containing homologues of haustorially expressed secreted proteins (HESPs) 735, 417, and 379, respectively.

Most abundantly represented members of the secretome had no evident homologues in the UniProtKB database (Additional File 2), or even in PuccinialesDB. However, some highly expressed candidates showed similarities to sequences suspected to be involved in pathogenicity or host-pathogen interactions (HESPs and CFEM-containing proteins). To obtain an accurate overview regarding the expression profiles of the secretome, we associated S+ members to 15-bp tags from a serial analysis of gene expression (SAGE). We analyzed expression patterns at three time points during the infection process of *M. larici-populina*: twice during the pre-biotrophic stage (2 and 22 hours after inoculation), and once during the biotrophic stage (5 days after inoculation) (Feau et al., unpublished). The last two time points were also investigated on both susceptible and resistant hosts, i.e. during compatible and incompatible interactions. A quarter (182 unisequences) of the S+ members was confidently associated to a SAGE tag. An average linkage hierarchical clustering of SAGE patterns revealed strong stage specificities of the secretome (Figure 2), similar to observations made on *U. fabae *genes initially identified using the yeast signal sequence trap [58]. Genes expressed during pre-biotrophic growth were clearly turned off at later biotrophic stages, while others were induced only during the biotrophic stage. Global expression patterns were consistent with observations made in our libraries, with 60% of the represented haustorium-enriched library unisequences being upregulated during the biotrophic stage. Compatible and incompatible treatments were grouped together at each time point, and the pre-biotrophic stage libraries were clustered together and separated from the biotrophic stage libraries. Even though similar stage specificity has been observed for the non-secreted counterpart (data not shown), the abundance of tags pointed towards higher expression levels for secreted proteins during the pre-biotrophic stage when compared to the rest of the transcriptome. On the other hand, secretome members found during the biotrophic stage had expression levels similar to their non-secreted counterpart (Additional File 3). The mean number of tags per unisequences correlated well with the abundance of clones per contig. While the haustorium-enriched library shared a somewhat similar number of genes encoding putative secreted proteins when compared to other libraries, their level of expression, as reflected by the percentage of identified sequences, was quite lower (Additional File 4). Secreted proteins accounted for 25% of *ex planta *libraries (from 15.3 to 33.0%), while it was only 6.8% for the haustorium-enriched library. Secretory activity among obligate biotrophs thus appears limited and strongly regulated. A strict control of the secretory activity is required to form the interface layers that are observed in biotrophic interactions [59] and this could be a component of the pathogen's strategy to evade recognition by host factors [19].

### Evolutionary constraints of the poplar leaf rust secretome: adaptive evolution

**Figure 3:**
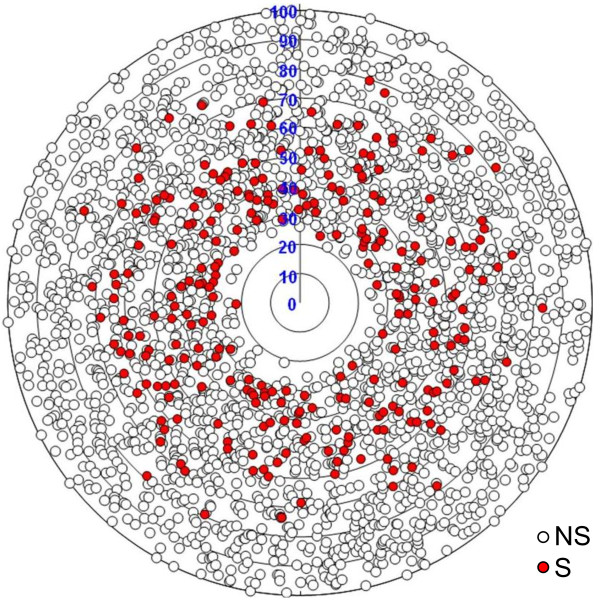
**Accelerated sequence divergence of secreted proteins between predicted ORFs from the *Melampsora *unisequence dataset and the *Puccinia graminis *f. sp. *tritici *gene models**. The set of *Melampsora *predicted ORFs (2319 non-secreted ORFs, represented by white circles; and 267 secreted ORFs, represented by red circles) was compared with translated gene models of *Puccinia graminis *f. sp. *tritici*. Each radius ranges from 0 (center) to 100 (outer circle), representing BLASTP percent identity. A BLASTP hit was considered significant when E-value ≤ 1e-4. Position of ORFs along the circumference is random.

None of the effectors described from various fungi are known to have close homologues beyond species or genus boundary [27]. In order to determine if the evolutionary model of poplar leaf rust S+ proteins was confined to the presence/absence pattern observed above, we generated new BLASTP searches using 6033 (5349 NS/684 S+) predicted ORFs against the translated gene models of *P. graminis *f. sp. *tritici*, and plotted the percentage of identity of each dataset to their closest homologues (Figure 3). Around 40% of each dataset had homologues in the *P. graminis *f. sp. *tritici *genome, a proportion that could be explained by the relatively short length of many ORFs, the presence of false ORFs predicted from non-coding regions such as UTRs, and the relative divergence expected between these genera [60]. However, approximately half of NS-predicted ORFs had high identity values (more than 70%) with their closest homologue from the *P. graminis *f. sp. *tritici *gene models compared to only 7% of S+-predicted ORFs, thus suggesting that the secretome is under different evolutionary constraints. Again, fast evolution of coding regions due to molecular arms races between pathogens and hosts might explain the high divergence between orthologues or the absence of close homologues in related species.

We used reciprocal TBLASTX searches (E-value ≤ 1e-30) among our taxonomic cDNA libraries to identify orthologues and/or paralogues in different *Melampsora *species libraries and to classify them into homologous gene groups (HGGs). In order to increase the number of HGGs, we included the gene models from *P. graminis *f. sp. *tritici*. We found 369 HGGs consisting of at least three different sequences with a minimum of two *Melampsora *unisequences retrieved. These HGGs included 1159 *Melampsora *unisequences and 437 *P. graminis *f. sp. *tritici *gene models. HGGs were divided to 283 non-secreted HGGs (76.7%) and 86 secreted HGGs (23.3%) according to their predicted localization. Consistent with the above similarity searches, approximately 45% of secreted HGGs had no *P. graminis *f. sp. *tritici *homologues (at E-value ≤ 1e-30), compared with 12% for non-secreted HGGs. Moreover, the proportion of secreted HGGs (23.3%) was twice the proportion of S+ unisequences (11.4%), and the mean number of *Melampsora *unisequences per HGG was slightly higher in secreted HGGs (4.3 unisequences/HGG) compared with non-secreted HGGs (2.9 unisequences/HGG). These results could suggest that a larger proportion of allelic forms and/or paralogue families exist among the rust secretome, which is consistent with an extensive sequence diversification motivated by the coevolutionary arms race [30].

**Figure 4:**
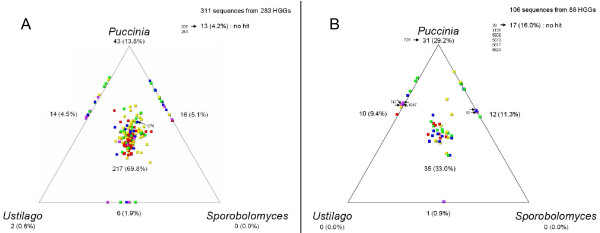
**Similarity of *Melampsora *homologous gene groups (HGGs) to the proteomes of other basidiomycetes**. Similarity of *Melampsora *A) non-secreted and B) secreted homologous gene groups (HGGs) to the proteomes of *Puccinia graminis *f. sp. *tritici*, *Ustilago maydis *and *Sporobolomyces roseus*. For each of the 369 *Melampsora *HGGs, a BLASTX search was performed against the proteomes of the other three species and SimiTri was used to plot the sequence similarity relationships between 417 consensus sequences derived from these HGGs and related species. Each tile in the graphics represents a unique consensus sequence and its relative position is computed from the raw BLAST scores derived above (with a cutoff of > 40). Hence, each tile's position indicates its degree of sequence similarity to each of the three selected databases. Sequences showing similarity to only one database are not shown. Sequences showing sequence similarity to only two databases appear on the lines joining the two databases. The position of positively selected HGGs is indicated by arrows. Tiles are colored by their highest BLASTX score for each of the databases: red ≥ 300; yellow ≥ 200; green ≥ 150; blue ≥ 100; and purple < 100.

In order to visualize the evolutionary relationships between *Melampsora *and other fungi we used SimiTri [61], which plots in two-dimensional space the relative similarities of gene sequences between one group (*Melampsora*) and three comparators. For each sequence included in an HGG, a BLASTX was performed against three other basidiomycetes for which genome sequences were available: *Puccinia *(*Pucciniomycotina*; *Pucciniomycetes*), the wheat stem rust, which is phylogenetically close to *Melampsora *and shares a similar biotrophic lifestyle; *Sporobolomyces *(*Pucciniomycotina*; *Microbotryomycetes*), a free-living saprobic yeast that is phylogenetically close to rusts but differs in its saprophytic lifestyle; and *Ustilago *(*Ustilaginomycotina*; *Ustilaginomycetes*), the corn smut, which is phylogenetically more distant from *Melampsora *but is also a plant pathogen. In a few cases, sequences within HGGs matched different paralogous sequences in one of the comparators (usually *Puccinia*). Consensus sequences were thus created from sequences having identical BLASTX hits in the three comparators, allowing multiple consensus sequences for a single HGG. SimiTri was used to plot 417 consensus sequences from 369 *Melampsora *HGGs against related species' gene models (Figure 4). For each HGG, most *Melampsora *sequences grouped slightly towards *Puccinia*, its closest relative (Figure 4). Again, the difference between non-secreted and secreted HGGs was striking with 69.8% of non-secreted HGGs exhibiting homologous sequences in the three comparators, while only 33.0% of secreted HGGs had such homologues. Even tough the percentage of secreted HGGs with no hit in the three other species (16.0%) was almost four times the percentage of unique non-secreted HGGs (4.2%), this was not sufficient to explain this difference. The percentage of HGGs unique to *Pucciniales *was twice higher for secreted HGGs (29.2% compared with 13.8%). Similar secreted/non-secreted HGGs ratios were observed for the percentage of secreted HGGs absent from *Ustilago *(11.3% compared with 5.1%) or from *Sporobolomyces *(9.4% compared with 4.5%). While HGGs absent from *Ustilago *but found in both *Puccinia *and *Sporobolomyces *could have been eliminated following the *Ustilaginomycotina*/*Pucciniomycotina *radiation, HGGs absent from the free-living *Sporobolomyces *but found in the more distant *Ustilago *could represent genes involved in host-pathogen interactions and/or biotrophic lifestyles. Similarly, recent observations on the whole genome sequences of *Ustilago maydis *and *Laccaria bicolor *suggested that the inventory of certain enzymes underwent massive gene loss as a result of its adaptation to a biotrophic (*U. maydis*) or symbiotic lifestyle (*L. bicolor*) [40,62].

**Figure 5:**
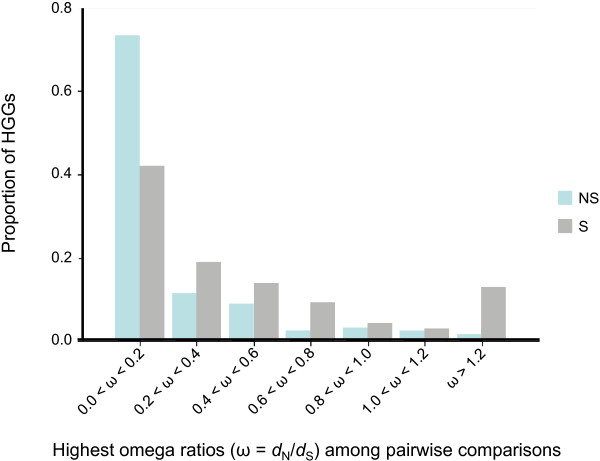
**Elevated *d*_N_/*d*_S _ratios among *Melampsora *homologous gene groups (HGGs) encoding secreted proteins**. The rates of non-synonymous nucleotide substitutions per non-synonymous site (*d*_N_) and the rates of synonymous nucleotide substitutions per synonymous site (*d*_S_) were calculated across all possible pairwise comparisons within each of the 369 HGGs using the maximum likelihood method implemented in the codeml program (runmode = -2) in the PAML 4 software package. The distribution of highest calculated ω (*d*_N_/*d*_S _ratios) among all pairwise comparisons within each HGG is shown. In cases where *d*_N _> 0 and *d*_S _= 0 (i.e. ω = ∞), the second higher ratio was selected.

Molecular genetic analysis of plant-pathogen interactions includes many layers of antagonistic coevolution. Investigation of molecular evolution at these various levels usually reveals diversifying selection and the selective maintenance of variation, resulting in positive selection at the genomic interfaces of escalating attack and defense systems [63]. Following this idea, one of the most reliable indicators of positive selection at the molecular level is a higher non-synonymous nucleotide substitution rate (*d*_N_) than the synonymous nucleotide substitution rate (*d*_S_) between two protein-coding DNA sequences (ratio ω = *d*_N_/*d*_S _> 1) [64]. Based on this criterion, statistical methods, such as the approximate (counting) and the maximum likelihood (ML) methods, have been developed [65-67]. We calculated the *d*_N _and *d*_S _rates across all possible pairwise sequence comparisons within each of the 369 HGGs using the ML method. The distribution of omega ratios (ω) was skewed towards extreme low values for non-secreted HGGs, with almost 75% of highest pairwise estimates of ω < 0.2 and only 5% > 0.8 (Figure 5). This distribution was quite different for secreted HGGs, with proportions around 40% and 20% of highest pairwise estimates of ω < 0.2 and > 0.8, respectively. For 13 HGGs, the *d*_N _value was significantly greater than *d*_S _(ω = *d*_N_/*d*_S _> 1.2) in at least one pairwise comparison (Table 3). Ten of these groups under positive selection were secreted HGGs (corresponding to 12% of the total secreted HGGs). Using a different approach based on Fixed-Effect Likelihood (FEL) statistics, Feau and colleagues detected positive selection in seven gene groups (including five putative secretome members) from a similar dataset [68]. Two of these seven gene groups corresponded to secreted HGGs unravelled using the *d*_N_/*d*_S _approach described here (92 and 6067, corresponding to MEL_49 and MEL_55, respectively, in [68]). Interestingly, eight positively selected secreted HGGs were found to encode cysteine-rich proteins with an even number of Cys residues that may be involved in disulfide bonds. An even number of Cys residues is generally indicative of the presence of disulfide bonds, which are formed between the thiol groups of cysteines. Disulfide bonds play an important role in the folding and stability of some proteins, usually proteins secreted to the extracellular medium, and are typical features of a subset of fungal and oomycete effectors, especially those acting in the plant apoplast [11,27].

**Table 3:** Characteristics of the *Melampsora *homologous gene groups (HGGs) predicted to be under positive selection (at least one pairwise ω = *d*_N_/*d*_S _> 1.2)

HGG	NS/S^a^	Length	BLASTX Fungi UniProtKB	E-value	BLASTX *Puccinia*	E-value	PFAM	Cys	ω^b^
205	NS	115	No hit		No hit		-	ND	2.68
254	NS	200-202	No hit		PGTT03363	1e-10	-	ND	2.59
1278	NS	173-175	XP_001211022 conserved hypothetical protein [*Aspergillus terreus*]	3e-07	PGTT11073	1e-34	-	ND	1.88
28	S	161	No hit		No hit		-	0	1.38
92	S	271-276^c ^(227)^d^	AAS45284 proline-rich antigen [*Chrysosporium lucknowense*]	4e-09	PGTT01207	2e-13	CFEM	6 (2)^d^	1.43
729	S	269	EAT81533 hypothetical protein [*Phaeosphaeria nodorum*]	7e-11	PGTT12331	7e-61	-	8	1.32
747	S	163	XP_757360 hypothetical protein [*Ustilago maydis*]	1e-04	PGTT02151	2e-14	-	4	1.95
4191	S	129	No hit		No hit		-	4	1.57
5606	S	119-123^e^	No hit		No hit		-	6	1.76
5610	S	98	No hit		No hit		-	6^g^	4.31
5617	S	168-169^f^	No hit		No hit		-	0^g^	1.23
5624	S	176	No hit		No hit		-	8	1.93
6067	S	285	XP_758577 hypothetical protein [*Ustilago maydis*]	6e-06	PGTT13234	5e-21	-	10^g^	1.27

BLASTX and PFAM hits were considered significant when E-value ≤ 1e-4 and 1e-5, respectively.

^a^NS: non-secreted; S: secreted.

^b^Highest calculated *d*_N_/*d*_S _ratio among all pairwise comparisons within each HGG.

^c^Length is 271 (*M. medusae *f. sp. *deltoidae*, *M. medusae *f. sp. *tremuloidae *and *M. occidentalis*), and 276 (*M. larici-populina*); an 86 amino acids insertion and a 5 amino acids minisatellite are also present in a *M. medusae *f. sp. *deltoidae *unisequence (length: 357) and a *M. larici-populina *unisequence (length: 281), respectively.

^d^A 44 amino acids deletion (including 4 Cys residues) is present in a *M. medusae *f. sp. *deltoidae *unisequence.

^e^Length is 119 (*M. medusae *f. sp. *tremuloidae*) and 123 (*M. occidentalis*).

^f^A 12 amino acids minisatellite is present in a *M. occidentalis *unisequence (length: 181).

^g^Additional Cys residues are present before the signal peptide cleavage site.

To identify additional HGGs under positive selection and detect the amino acid residues that are under positive selection, we contrasted the M2A/M1A, M8/M7 and M8/M8A models with likelihood ratio tests (Additional Files 5 and 6; see Methods) [64,66]. Significant evidence of positive selection was found in 4 (including 2 secreted HGGs) of the 369 HGGs. Selective pressures had previously been identified for one of these secretome members using FEL statistics and a population genetics approach [68,69]. This particular secreted HGG has homology with HESP-417 from *M. lini*, a gene known to be expressed in haustoria and encoding a secreted protein with an even number of Cys residues [26].

We assessed the position of positively selected HGGs on above SimiTri plots (indicated by arrows, Figure 4): only one non-secreted HGG (1278) had homologues in the three databases, two secreted HGGs (747 and 6067) were absent from *Sporobolomyces*, one secreted HGG (92) was absent from *Ustilago*, one secreted HGG (729) was present only in *Puccinia*, and the remaining HGGs (6 secreted and 2 non-secreted HGGs) had no hit. Two other groups of sequences identified using FEL statistics [68] had corresponding HGGs plotted on the line between *Puccinia *and *Ustilago *(absent from *Sporobolomyces*) (data not shown).

## Conclusion

Database searches with sequences of small secreted proteins from fungi commonly do not yield homologues or known protein domains, the only recognizable features being the presence of a signal peptide for secretion and, in many cases, an even number of cysteine residues. Despite these commonalities, effectors appear to be evolutionarily diverse and highly variable in their distribution, showing very limited phylogenetic distributions possibly due to accelerated evolution stimulated by plant-pathogen arms races [27]. In a straightforward *in silico *approach, we generated a first overview of the secretome from poplar leaf rusts belonging to the genus *Melampsora*, unravelling an unknown and diversifying set of genes. The identification of positive selection in putative secreted proteins reported here suggests that these genes are likely to encode candidate effectors implicated in host-pathogen interactions. Such information should be used to augment other selection criteria (such as gene expression data) for prioritizing candidate effector genes for functional studies. Two intriguing properties of rust fungi are their host specificity and their need for host alternation. Even though host specificity is probably controlled at several levels, examples from the flax rust fungus suggest that the secretion of effectors plays a prominent role [26]. Their intimate interactions with host factors expose them to very strong selective pressures resulting in their rapid evolutionary turnover. However, poplar leaf rust effectors not only cope with the poplar defense machinery, they also face another phylogenetically diverse host plant, which differs between species, from other dicots to monocots and even gymnosperms. Is this particularity responsible for a greater sequence diversification? Or is it responsible for a broader arsenal of effectors in poplar leaf rusts? The recent completion of the *M. larici-populina *genome http://genome.jgi-psf.org/Mellp1/Mellp1.home.html will reveal further information on the whole repertoire of secreted proteins in this pathogen. Comparative genomics studies with other biotrophs should elucidate molecular mechanisms underlying common strategies to infect plants. The identification of host targets will provide further insight into the evolutionary forces that shaped the rust secretome, a key step facilitated by the availability of the poplar genome sequence [70] and transcript profiling of poplar-rust interactions [55,71,72]. This pathosystem clearly represents an unprecedented opportunity to understand the particularities of host-pathogen interactions.

## Methods

### Rust and plant material

cDNA libraries were constructed from *ex planta *material (resting and germinating urediniospores, germ tubes, etc.) of four different *Melampsora *taxa. Fungal materials from isolates of the North American subspecies *M. medusae *f. sp. *deltoidae *and the Eurasian species *M. larici-populina *were harvested from naturally infected leaves of eastern cottonwood (*Populus deltoides*) and hybrid poplar (*P. balsamifera *× *P. maximowiczii*) clones, respectively. *Melampsora medusae *f. sp. *tremuloidae *and *M. occidentalis *mono-uredinial cultures were used to inoculate fresh leaves of *P. tremuloides *and *P. trichocarpa*, respectively. Inoculated leaves were maintained for 13 days in a growth chamber at 60% humidity, 19°C and 16 h photoperiod. In addition, we generated one additional haustorium-enriched library (biotrophic stage) for the *M. larici-populina *species. Haustoria were isolated by affinity chromatography as described by Hahn and Mendgen [73]. A mixture of plant leaf and fungal tissues was collected 6 days after inoculation of a rust-susceptible *P*. × *jackii *clone 1014 with the rust strain *Mlp *Berth. 3729. A 100-μm pore size nylon mesh was used to remove the bulk of the plant cell material from the crude preparation, which was then passed through an 11-μm pore size nylon mesh to remove intact plant cells. An affinity column was prepared by covalently attaching concanavalin A (Pharmacia Biotech) to cyanogen bromide-activated Sepharose 6 MB (Pharmacia Biotech) as described in the manufacturer's protocol. Samples of purified haustoria colored using Calcofluor white (50 μM final concentration) were then examined using a fluorescence microscope under UV filters.

### cDNA libraries and DNA sequencing

Haustoria samples were pelleted by centrifugation at 20,800 g, washed twice with sterile distilled water, and maintained at -80°C until total RNA extraction. Fungal material was directly frozen in liquid nitrogen and ground using the Mixer Mill MM 300 with 2 mm tungsten carbide beads. All the following manipulations were performed according to the manufacturer's instructions. Total RNA was extracted from *ex planta *material and purified haustoria using the RNeasy mini kit (Qiagen, Valencia, CA, USA) and the Absolutely RNA^® ^miniprep kit (Stratagene, La Jolla, CA, USA), respectively. PolyA RNA was purified using biotylinated oligo-dT/streptavidin-coated magnetic beads (Dynabeads^® ^Oligo (dT)_25_; Dynal Biotech, Oslo, Norway). Haustorium-enriched *M. larici-populina *as well as *M. medusae *f. sp. *tremuloidae *and *M. occidentalis ex planta *cDNA libraries were generated using the SMART™ cDNA library construction kit (Clontech, Mountain View, CA, USA). *Melampsora larici-populina *and *M. medusae *f. sp. *deltoidae ex planta *cDNA libraries were constructed using the pBlueScript II XR cDNA library construction kit (Stratagene) according to the manufacturer's instructions. Following the column sepharose chromatography step included in the protocol, only size fractions above 500 bp were retained for ligation in the *Sfi*I-digested, dephosphorylated pDNR-LIB vector. Plasmid ligations were transformed by electroporation into *E. coli *ElectroMAXTM DH10BTM Cells (Invitrogen, Carlsbad, CA, USA). After library amplification and tittering, individual colonies were transferred onto 384-well microtiter plates containing LB medium with 30 μg/ml chloramphenicol for PCR amplification and sequencing. cDNA inserts were amplified according to the manufacturer's PCR protocol and then sequenced with the M13 forward primer (5'-GTAAAACGACGGCCAGT-3') using the Big Dye Terminator Cycle Sequencing Kit v1.1 on an ABI 3730xl sequencer (Applied Biosystems) at the CHUQ Research Centre (CRCHUQ) Sequencing and Genotyping Platform, Quebec City, QC, Canada. Sequences were deposited at NCBI under accession numbers GW672673 to GW687576.

### cDNA assembly and ORF prediction

Raw sequences were trimmed and cleaned using the *Phred *software [74], which resulted in the identification and removal of poor quality regions (quality cut-off of 20). Cross-match was then used to mask vector sequence in each read (minimum match of 10, minimum score of 20). The extent of redundancy for each library was ascertained using the *Phrap *software (Phil Green, http://www.phrap.org), which was also used to compile the unisequence set (minimum match of 50, minimum score of 100). In order to identify and remove plant sequences in the ESTs, unisequences were used in BLAST comparisons against the *Populus trichocarpa *genome and predicted gene models. Initial ORF prediction for *Melampsora *spp. was generated with the bestORF algorithm (Softberry) using *Ustilago *parameters.

### Sequence analysis

Similarity searches for full length sequences and conserved domains were performed using a combination of standard bioinformatics programs and customized Python scripts. Each assembled transcript was searched against UniProtKB database (Release 15.5, TrEMBL and Swiss-Prot at http://www.uniprot.org) resources [75] using the BLASTX algorithm [76]. Using UniProt/Gene Ontology (GO) crossed tables, candidate GO assignments were predicted on the basis of best transcripts matches (E-value <10^-05^) to the UniProt reference sequences. Categories were assigned on the basis of the biological, functional and molecular annotations available from GO http://www.geneontology.org[46].

Additionally, we constructed and searched (using the BLASTX algorithm) two other fungal sequence databases. The BasidiomycotaDB database included *Basidiomycota *(excluding *Pucciniales*) protein sequences from the non-redundant database of NCBI (124,751 sequences), a 6-frame translation of *Basidiomycota *(excluding *Pucciniales*) EST sequences from NCBI (287,259 sequences), and gene models from eight genome projects: *Coprinus cinereus *Okayama 7 (#130) (Broad Institute); *Cryptococcus neoformans *var. *grubii *serotype A, strain H99 (Broad Institute); *Laccaria bicolor *S238N-H82 (JGI); *Malassezia globosa *CBS 7966 (Procter and Gamble Co.); *Phanerochaete chrysosporium *RP78 (JGI); *Postia placenta *MAD-698 (JGI); *Sporobolomyces roseus *IAM 13481 (JGI); and *Ustilago maydis *521 (Broad Institute) (a total of 85,025 gene models). The PuccinialesDB database included *Pucciniales *protein sequences from the non-redundant database of NCBI (390 sequences), a 6-frame translation of *Pucciniales *EST sequences from NCBI (84,006 sequences), and gene models from *P. graminis *f. sp. *tritici *CRL 75-36-700-3 (20,567 gene models). The *hmmpfam *program (HMMer software; http://hmmer.janelia.org[77] was used to search the PFAM HMM profile database of protein domains [45].

### Signal peptide prediction

*In silico *predictions of secreted proteins were carried out using a combination of SignalP 3.0, TargetP 1.1 and TMHMM 2.0 [44,78,79]. The SignalP algorithms incorporate a cleavage site and signal peptide prediction based on artificial neural networks (NN) and hidden Markov models (HMM). In order to support the SignalP results and exclude proteins with either mitochondrial targeting peptides or transmembrane domains, protein sequences were also entered into different prediction servers. TargetP is a neural networks server that predicts the subcellular localization of eukaryotic proteins based on the presence of any of the N-terminal presequences, either chloroplast transit peptide (for plant predictions), mitochondrial targeting peptide or secretory pathway signal peptide, while TMHMM uses hidden Markov models for the prediction of transmembrane helices. Following predictions, the output files were manipulated to select signal peptides containing sequences using the following criteria: (1) positive SignalP-HMM Sprob score, (2) positive SignalP-NN Smax and D scores, (3) TargetP signal peptide prediction, and (4) no transmembrane domains. SignalP-HMM Sprob score was selected because of its ability to discriminate between N-terminal signal peptides and N-terminal signal anchors, while SignalP-NN Smax and D scores are the most accurate single scores [80]. Furthermore, because TMHMM may not distinguish signal peptides from transmembrane domains, deduced proteins with a single transmembrane domain within 40 amino acids of the N-terminus were also considered as potential secreted proteins.

### SAGE analyses

The SAGE method was used as initially described in Velculescu et al. [81,82] at the CHUQ Research Centre (CRCHUQ) SAGE Platform, Quebec City, QC, Canada http://www.crchuq.ulaval.ca/plateformes/gpb. Fungal materials from *M. larici-populina *strain *Mlp *Berth. 3729 were harvested from both susceptible (*P*. × *jackii*) and resistant (*P. trichocarpa *× *P. deltoides *'Boelare') hosts at two different time points (22 hours and 5 days) after inoculation. Additionally, germinating urediniospores and germ tubes were collected 2 hours after inoculation on the susceptible cultivar by painting leaves with 5% cellulose acetate (dissolved in acetone), letting the acetone evaporate, and stripping the cellulose acetate film off the leaves. For each of the five treatments, 50 micrograms total RNA were extracted using the RNeasy mini kit (Qiagen, Valencia, CA, USA) and poly(A) RNA isolated with the mRNA Mini kit (Qiagen), annealed with the biotin-50-T18-30 primer, and converted into cDNA using the cDNA synthesis kit (Invitrogen, Carlsbad, CA, USA). The resulting cDNA were digested with *Nla*III (anchoring enzyme), and the 3' restriction fragments were isolated with streptavidin-coated magnetic beads (Dynal Biotech, Oslo, Norway) before being separated into two populations. Each population was ligated to one of the two annealed linker pairs and washed to remove unligated linkers. The tag beside the most 3' *NIa*III restriction site (CATG) of each transcript was released by digestion with *Bsm*FI (tagging enzyme). The blunting kit from Takara Co. (Otsu, Japan) was used for the blunting and ligation of the two tag populations. The resulting ligation products containing the ditags were amplified by PCR with an initial denaturation step of 1 min at 95°C, followed by 22 cycles of 20 sec at 94°C, 20 sec at 60°C and 20 sec at 72°C with 27 bp primers. The PCR products were then digested with *Nla*III and the band containing the ditags was extracted from 12% acrylamide gel. The purified ditags were self-ligated to form concatemers of 500-1800 bp isolated by agarose gel. The resulting DNA fragments were ligated into the *Sph*I site of pUC19 and cloned into UltraMAX DH5aFT *E. coli *cells (Invitrogen, Carlsbad, CA, USA). White colonies were screened by PCR to select long inserts for automated sequencing as previously described for cDNA libraries.

Sequence files were analyzed using the SAGEparser program [83]. Tags corresponding to linker sequences were discarded and duplicate concatemers were counted only once. To identify the transcripts, the sequences of 15 bp SAGE tags (*Nla*III site CATG plus adjacent 11 bp tags) were matched with a collection of unassembled poplar and *M. larici-populina *ESTs with polyA tail using a customized Python script. To avoid the possibility of sequencing errors in the EST database, the matches that were identified only once among the EST database were not considered.

### Positive selection analyses

Homologous gene groups (HGGs) were identified from reciprocal TBLASTX searches (E-value < 1e-30) between libraries and *P. graminis *f. sp. *tritici *gene models followed by graph clustering analysis using a TCL implementation of the Deep-First Search algorithm. Each HGG included at least two sequences from *Melampsora *unisequences and a minimum of three total sequences. HGGs were either removed or divided when overlapping regions where too short or when similarity was not found throughout the majority of the unisequence coding sequences. Furthermore, sequences with gaps across the aligned coding sequences were removed in order to minimize the impact of the pitfalls of positive selection analyses, such as gap-induced misalignments and relaxed selection in pseudogenes. The resulting 369 HGGs were then submitted to positive selection analyses using a suite of program grouped into a single Python script. The protein sequences in each HGG were first aligned with ClustalW [84] and the corresponding coding DNA sequences were automatically extracted. A Neighbor-joining phenetic tree based on distance matrix between nucleotidic sequences was then reconstructed for each HGG and used as starting tree for Bayesian inference and Markov Chain Monte Carlo simulations (B/MCMC) (only possible with HGG of 4 sequences and more; the Neighbor-joining tree reconstructed with PAUP* was used for HGG of 3 sequences). Prior to the B/MCMC, the models for nucleotide substitutions were selected using the hierarchical likelihood ratio test (hLRT) implemented in the Modeltest 3.7 program. Adaptive evolution was first estimated by pairwise calculation of the rates of nonsynonymous nucleotide substitutions per nonsynonymous site (*d*_N_) and the rates of synonymous nucleotide substitutions per synonymous site (*d*_S_) between all members of an HGG. Additionally, as adaptive evolution is likely to act on a small subset of amino acid residues and hence averages of substitution rates across the gene may not strictly indicate positive selection, HGGs were scanned for adaptive evolution using codon-based substitution models that allow ω to vary among sites, with the parameters of the model estimated using maximum likelihood. These analyses were conducted using the codeml application from the PAML package version 4 [67]. Bayesian inference of phylogeny aimed at estimating the posterior probabilities and branch length of phylogenetic trees as starting values for codeml maximum likelihood iteration under codon model M0 to get fixed branch lengths. The resulting ω pairwise calculations are shown in Table 3 for all HGGs with at least one pairwise calculation showing ω > 1.2. We also contrasted the codon substitution models M1A (neutral), M2A (selection), M7 (beta), M8 (beta and ω) and M8A (beta and ω = 1; [85]). The model M1A assumes two site classes in proportions p0 and p1 = 1-p0 with 0 < ω 0 < 1 (conserved) and ω 1 = 1 (neutral). M2A adds an additional class of site with ω 2 as a free parameter, allowing for sites with ω 2 > 1 with proportion p2. Model M7 uses a beta distribution of sites within the interval 0 < ω < 1. M8 adds an extra class of sites to the M7 model, allowing for positively selected sites with ω > 1, while this extra *d*_N_/*d*_S _category is restricted to one in model M8A [85]. From these models, statistical significance was tested using likelihood ratio tests by comparing the null models M1A, M7 and M8A with the alternative M2A, M8 and M8 models, respectively. Models M2A and M8 are tests of positive selection among codon sites and were implemented with at least three different starting ω values (0.2, 1.0 and 2.0). Twice the difference in log likelihood ratio between null and alternative models was compared with a χ2 distribution with two degrees of freedom. The likelihood ratio tests assess whether the alternative models fits the data better than the null model and is known to be conservative in simulation tests. For HGGs that tested positive using the ML method, posterior Bayesian probabilities of site classes were inferred for each amino acid site by using the Bayes empirical Bayes method [86].

## Authors' contributions

DLJ contributed to the conception and design of the project, conducted laboratory experiments (EST libraries), designed bioinformatics scripts, performed analysis and interpretation of the data and drafted the manuscript. NF and RCH helped conceive the project and participated in its coordination. NF conducted laboratory experiments (EST libraries and SAGE analysis) and helped design bioinformatics scripts. NF, PT and RCH critically revised the manuscript. All authors read, helped to edit, and approved the final manuscript.

## Supplementary Material

Additional file 1 **PFAM domains found in the putative secreted members of the *Melampsora *unisequence dataset**. Domains represented in more than five unisequences are shown. PFAM hits were considered significant when E-value ≤ 1e-5. Click here for file

Additional file 2 **Top five most abundantly represented secretome members in each *Melampsora *library**. BLASTX hits were considered significant when E-value ≤ 1e-4. Click here for file

Additional file 3 **Mean number of SAGE tags associated with *Melampsora *unisequences**. Click here for file

Additional file 4 **Proportion of contigs and clones included in the putative secretome of *Melampsora***. Click here for file

Additional file 5 **Characteristics of the *Melampsora *homologous gene groups (HGGs) predicted to be under positive selection (site-based analysis with codeml)**. BLASTX and PFAM hits were considered significant when E-value ≤ 1e-4 and 1e-5, respectively. Click here for file

Additional file 6 **Significant likelihood ratio tests and sites under positive selection as inferred under the site class models M1A, M2A, M7, M8 and M8A of codeml applied to each of the *Melampsora *homologous gene groups (HGGs)**. Click here for file
