# Supplementary material for: Comparative analysis of secreted protein evolution using expressed sequence tags from four poplar leaf rusts (Melampsora spp.)
Source: BMC Genomics. 2010 Jul 8;11:422. doi: 10.1186/1471-2164-11-422 (PMC2996950; doi:10.1186/1471-2164-11-422)
Supplement: Additional file 1 — PFAM domains found in the putative secreted members of the Melampsora unisequence dataset. Domains represented in more than five unisequences are shown. PFAM hits were considered significant when E-value ≤ 1e-5. [file 1471-2164-11-422-S1.DOC]

## Additional file 1 – PFAM domains found in the putative secreted members of the Melampsora unisequence dataset.

| PFAM domain (PFAM accession No.) | # *M. larici-populina* haustoria unisequences  (# clones) | # *M. larici-populina*  *ex planta* unisequences  (# clones) | # *M. medusae* f.s p. *deltoidae*  *ex planta* unisequences  (# clones) | # *M. medusae* f.s p. *tremuloidae* *ex planta* unisequences  (# clones) | # *M. occidentalis*  *ex planta* unisequences  (# clones) | Total |
| --- | --- | --- | --- | --- | --- | --- |
| CFEM^a^ (PF05730) | 1 (1) | 3 (34) | 5 (82) | 6 (43) | 7 (39) | 22 (199) |
| Glyco_hydro_16^a^ (PF00722) |  | 1 (36) | 1 (2) |  |  | 2 (38) |
| Peptidase_S8^a^ (PF00082) |  | 2 (12) ^b^ | 2 (6) ^c^ |  |  | 4 (18) |
| Subtilisin_N (PF05922) |  | 1 (10) ^b^ | 2 (6) ^c^ |  |  | 3 (16) |
| Asp^a^ (PF00026) |  | 1 (2) | 2 (10) |  |  | 3 (12) |
| Polysacc_deac_1^a^ (PF01522) |  | 1 (6) |  | 1 (4) | 1 (1) | 3 (11) |
| Thaumatin^a^ (PF00314) | 1 (2) | 1 (2) | 1 (1) | 1 (1) | 1 (3) | 5 (9) |
| SLT^a^ (PF01464) |  | 1 (5) |  |  |  | 1 (5) |

Domains represented in more than five unisequences are shown. PFAM hits were considered significant when E-value ≤ 1e-5.

^a^Domains only found in the S+ dataset.

^b^Peptidase_S8 and Subtilisin_N domains are shared in 1(10).

^c^Peptidase_S8 and Subtilisin_N domains are shared in 1(4).
