# Supplementary material for: Comparative analysis of secreted protein evolution using expressed sequence tags from four poplar leaf rusts (Melampsora spp.)
Source: BMC Genomics. 2010 Jul 8;11:422. doi: 10.1186/1471-2164-11-422 (PMC2996950; doi:10.1186/1471-2164-11-422)
Supplement: Additional file 2 — Top five most abundantly represented secretome members in each Melampsora library. BLASTX hits were considered significant when E-value ≤ 1e-4. [file 1471-2164-11-422-S2.DOC]

Additional file 2 - Top five most abundantly represented secretome members in each Melampsora library.

| Contig^a^ | Redundancy^b^ | BLASTX against Fungi UniProtKB | E-value |
| --- | --- | --- | --- |
| *M. larici-populina* haustoria | | |  |
| 1. Contig601 (14) | 11 | No hit | - |
| 2. Contig599 (16) | 10 | No hit | - |
| 3. Contig597 (17) | 9 | No hit | - |
| 4. Contig585 (28) | 7 | No hit | - |
| 5. Contig582 (30) | 6 | No hit | - |
| *M. larici-populina* *ex planta* | | |  |
| 1. Contig282 (1) | 121 | ref|XP_445223.1| unnamed protein product [*Candida* *glabrata*] | 4e-05 |
| 2. Contig281 (2) | 110 | No hit | - |
| 3. Contig278 (4) | 65 | ref|XP_445223.1| unnamed protein product [*Candida* *glabrata*] | 4e-05 |
| 4. Contig275 (7) | 50 | ref|XP_566697.1| hypothetical protein [*Cryptococcus* *neoformans* var. *neoformans*] | 7e-18 |
| 5. Contig271 (11) | 38 | gb|ABB96272.1| hesp-379 [*Melampsora* *lini*] | 9e-60 |
| *M. medusae* f. sp. *deltoidae* *ex planta* | | |  |
| 1.Contig456 (10) | 62 | ref|XP_001800355.1| hypothetical protein [*Phaeosphaeria* *nodorum*] | 2e-05 |
| 2. Contig455 (11) | 57 | gb|AAS45284.1| proline-rich antigen [*Chrysosporium* *lucknowense*] | 7e-10 |
| 3. Contig452 (14) | 54 | No hit | - |
| 4. Contig451 (15) | 51 | emb|CAA43289.1| priA [*Lentinula* *edodes*] | 1e-14 |
| 5. Contig450 (16) | 50 | ref|XP_566697.1| hypothetical protein [*Cryptococcus* *neoformans* var. *neoformans*] | 9e-13 |
| *M. medusae* f. sp. *tremuloidae ex planta* | | |  |
| 1.Contig638 (1) | 29 | No hit | - |
| 2. Contig637 (2) | 28 | No hit | - |
| 3. Contig635 (3) | 25 | No hit | - |
| 4. Contig634 (4) | 23 | No hit | - |
| 5. Contig633 (5) | 21 | No hit | - |
| *M. occidentalis* *ex planta* | | |  |
| 1.Contig520 (3) | 17 | No hit | - |
| 2.Contig519 (4) | 13 | ref|XP_001880124.1| predicted protein [*Laccaria* *bicolor*] | 1e-12 |
| 3. Contig518 (5) | 11 | gb|ABV46587.1| GPI anchored CFEM domain [*Monacrosporium* *haptotylum*] | 1e-09 |
| 4. Contig517 (6) | 11 | No hit | - |
| 5. Contig515 (8) | 11 | ref|XP_001800023.1| hypothetical protein [*Phaeosphaeria* *nodorum*] | 9e-60 |

BLASTX hits were considered significant when E-value ≤ 1e-4.

^a^Abundance position in the whole library is indicated in brackets.

^b^Number of clones assembled per contig.
