# Supplementary material for: Comparative analysis of secreted protein evolution using expressed sequence tags from four poplar leaf rusts (Melampsora spp.)
Source: BMC Genomics. 2010 Jul 8;11:422. doi: 10.1186/1471-2164-11-422 (PMC2996950; doi:10.1186/1471-2164-11-422)
Supplement: Additional file 3 — Mean number of SAGE tags associated with Melampsora unisequences. [file 1471-2164-11-422-S3.DOC]

## Additional file 3 - Mean number of SAGE tags associated with Melampsora unisequences.

| Library^a^ | Mean number of tag  associated with S+^b^ unisequences | Mean number of tag  associated with NS^c^ unisequences | Mean number of tag (S+ unisequences)  Mean number of tag (NS unisequences) |
| --- | --- | --- | --- |
| 2H | 70.6 | 26.4 | 2.7 |
| 22H_C | 86.7 | 27.9 | 3.1 |
| 22H_I | 99.6 | 33.2 | 3.0 |
| 5D_C | 15.9 | 10.6 | 1.5 |
| 5D_I | 3.4 | 4.9 | 0.7 |

^a^2H: 2 hours after inoculation (germinating spores); 22H_C: 22 hours after inoculation (compatible interaction); 22H_I: 22 hours after inoculation (incompatible interaction); 5D_C: 5 days after inoculation (compatible interaction); and 5D_I: 5 days after inoculation (incompatible interaction).

^b^S+: Final set of unisequences encoding putative secreted proteins, following reassignments based on reciprocal BLAST.

^c^NS: Final set of unisequences not predicted to encode putative secreted proteins.
