# Supplementary material for: Comparative analysis of secreted protein evolution using expressed sequence tags from four poplar leaf rusts (Melampsora spp.)
Source: BMC Genomics. 2010 Jul 8;11:422. doi: 10.1186/1471-2164-11-422 (PMC2996950; doi:10.1186/1471-2164-11-422)
Supplement: Additional file 4 — Proportion of contigs and clones included in the putative secretome of Melampsora. [file 1471-2164-11-422-S4.DOC]

## Additional file 4 - Proportion of contigs and clones included in the putative secretome of Melampsora.

| Library | % S+^a^ contigs | % S+ clones | _# S+ clones/contig_  # NS^b^ clones/contig |
| --- | --- | --- | --- |
| *M. larici-populina* haustoria | 6.3 | 6.8 | 0.8 |
| *M. larici-populina* *ex planta* | 11.7 | 33.0 | 2.4 |
| *M. medusae* f. sp. *deltoidae ex planta* | 14.0 | 22.9 | 1.4 |
| *M. medusae* f. sp. *tremuloidae ex planta* | 15.9 | 29.7 | 1.6 |
| *M. occidentalis* *ex planta* | 9.1 | 15.3 | 1.7 |

^a^S+: Final set of unisequences encoding putative secreted proteins, following reassignments based on reciprocal BLAST.

^b^NS: Final set of unisequences not predicted to encode putative secreted proteins.
