# Supplementary material for: Comparative analysis of secreted protein evolution using expressed sequence tags from four poplar leaf rusts (Melampsora spp.)
Source: BMC Genomics. 2010 Jul 8;11:422. doi: 10.1186/1471-2164-11-422 (PMC2996950; doi:10.1186/1471-2164-11-422)
Supplement: Additional file 5 — Characteristics of the Melampsora homologous gene groups (HGGs) predicted to be under positive selection (site-based analysis with codeml). BLASTX and PFAM hits were considered significant when E-value ≤ 1e-4 and 1e-5, respectively. [file 1471-2164-11-422-S5.DOC]

Additional file 5 - Characteristics of the Melampsora homologous gene groups (HGGs) predicted to be under positive selection (site-based analysis with codeml).

| HGG | NS/S^a^ | Length | BLASTX  Fungi UniProtKB | E-value | BLASTX  *Puccinia* | E-value | PFAM | Cys | ω |
| --- | --- | --- | --- | --- | --- | --- | --- | --- | --- |
| 39 | NS | 310^b^ | gb|EDR08463.1| predicted protein  [*Laccaria bicolor*] | 2e-122 | PGTT06188 | e-106 | Ribosomal 60S | ND | 0.06 |
| 72 | NS | 461 | emb|CAA51932.1| elongation factor  [*Puccinia graminis*] | 8e-69 | PGTT14858 | e-127 | GTP_EFTU | ND | 0.15 |
| 4243 | S | 135 | gb|ABB96273.1| hesp-417  [*Melampsora lini*] | 4e-09 | No hit |  | - | 4^d^ | 0.62 |
| 9030 | S | 391-395^c^ | ref|XP_001058922.1| MIR-interacting saposin-like  [*Rattus norvegicus*] | 3e-15 | PGTT04024 | 2e-08 | - | 3 | 0.36 |

BLASTX and PFAM hits were considered significant when E-value ≤ 1e-4 and 1e-5, respectively.

^a^NS: non-secreted; S: secreted.

^b^A 6 amino acids minisatellite is absent from a *M. medusae* f. sp. *deltoidae* unisequence (length: 304).

^c^A 36 amino acids deletion is present in a *M. occidentalis* unisequence (length: 357).

^d^An additional Cys residue is present before the signal peptide cleavage site in *M. medusae* f. sp. *tremuloidae* and *M. occidentalis*.
