# Supplementary material for: Comparative analysis of secreted protein evolution using expressed sequence tags from four poplar leaf rusts (Melampsora spp.)
Source: BMC Genomics. 2010 Jul 8;11:422. doi: 10.1186/1471-2164-11-422 (PMC2996950; doi:10.1186/1471-2164-11-422)
Supplement: Additional file 6 — Significant likelihood ratio tests and sites under positive selection as inferred under the site class models M1A, M2A, M7, M8 and M8A of codeml applied to each of the Melampsora homologous gene groups (HGGs). [file 1471-2164-11-422-S6.DOC]

## Additional file 6 - Significant likelihood ratio tests and sites under positive selection as inferred under the site class models M1A, M2A, M7, M8 and M8A of codeml applied to each of the Melampsora homologous gene groups (HGGs).

| HGG | -2ΔlnL (M2A vs. M1A) ^a^ | Positively selected sites (M2A) ^b^ | -2ΔlnL (M8 vs. M7) ^a^ | -2ΔlnL (M8 vs. M8A) | Positively selected codon (M8) ^b^ |
| --- | --- | --- | --- | --- | --- |
| 39 | 16.70***^c^ | **31M**, **57Q**, 65S, **99Q**, *100M* | 25.64*** | 19.86*** | **31M**, **57Q**, 65S, **99Q**, **100M**, 126S |
| 72 | 10.84*** | *203S*, 209E, 211A, 212T, 213S, **217S**, *218A*, 229A, **231E** | 12.12*** | 12.07*** | *203S*, 209E, **211A**, *212T*, 213S, **217S**, **218A**, *229A*, **231E** |
| 4243 | 8.37* | 17M**^4^**, *57K*, 71V, 98S, 108Q, 110D, *132T*, *133Y*, 134T | 8.49* | 8.36* | 17M**^d^**, 42S, *57K*, 71V, 98S, 108Q, *110D*, *132T*, *133Y*, 134T, 135G |
| 9030 | 6.02* | 67H, 107F, 108V, 110G, 150I, 159D, 196Q, *236**, 246D, 250K, 275A | 6.02* | 6.02* | 67H, 107F, 108V, 110G, 150I, 159D, *196Q*, **236***, 246D, 250K, 275A |

^a^Mean of likelihood ratio tests [-2(lnL alternative hypothesis - lnL null hypothesis)] with three different starting omega values (0.2, 1, 2.0).

^b^Positively selected sites by Bayes Empirical Bayes (BEB) inference with posterior probabilities P > 0.5-0.9: roman; 0.9-0.95: italics; 0.95-0.99: bold; 0.99-1: underlined bold.

^c^***: significant at the 0.1% level; **: significant at the 1% level; *: significant at the 5% level.

^d^17M is located before the signal peptide cleavage site.
